# Developing count regression techniques for predicting the number of new type 2 diabetes cases in Saudi Arabia

**DOI:** 10.1371/journal.pone.0341436

**Published:** 2026-01-23

**Authors:** Faten Al-hussein, Laleh Tafakori, Mali Abdollahian, Khalid Al-Shali

**Affiliations:** 1 School of Science, RMIT University, Melbourne, Victoria, Australia; 2 Department of Mathematics and Statistics, College of Sciences, University of Jeddah, Jeddah, Saudi Arabia; 3 Department of Medicine, King Abdulaziz University Hospital, Jeddah, Saudi Arabia; Cairo University, EGYPT

## Abstract

Type 2 diabetes (T2D) is a chronic condition affecting millions globally. A robust predictive model to estimate the number of new cases of T2D can facilitate precise monitoring and effective intervention strategies. This study aims to predict the number of new T2D cases per month in Saudi Arabia and identify the Key Performance Indicators (KPIs) associated with T2D, using count regression models, Poisson Regression (PR), Negative Binomial Regression (NBR), Poisson Inverse Gaussian Regression (PIGR), and Bell Regression (BR). De-identified data from 1,000 patients with T2D in Saudi Arabia were used to develop the models. The performance of the full models, which include recommended Key Performance Indicators (KPIs), is compared using metrics such as the coefficient of determination (R^2^), root mean squared error (RMSE), mean absolute error (MAE), 10-fold cross-validation (CV-10), Akaike information criterion (AIC), and Bayesian information criterion (BIC). The most significant KPIs identified by the full models were utilized to develop the reduced models. The full NBR model outperformed other models, achieving R² of 0.88, RMSE of 0.93, MAE of 0.69, CV-10 of 1.21, AIC = 873.23, and BIC = 880. The reduced NBR model, focusing solely on the five most influential variables (marital status, age, body mass index (BMI), total cholesterol (TC), and high-density lipoprotein (HDL)), with R² = 0.84, RMSE = 1.10, MAE = 0.86, CV-10 = 1.37, AIC = 899, and BIC = 910, also outperformed other reduced models. The Likelihood Ratio Test (LRT) did not show a significant difference between the full and reduced NBR models (p = 0.694), supporting the adequacy of the reduced model. The proposed reduced model, utilizing only five significant KPIs, can help healthcare providers develop effective, targeted strategies by monitoring a smaller number of KPIs to reduce the rising number of T2D cases in Saudi Arabia.

## 1 Introduction

Type 2 diabetes (T2D) is likely to double globally within the next two decades [1], leading to significant health and economic issues on a global level. The Middle East and North Africa regions are also expected to see a similar rise due to the rapid economic growth, urbanization, and changes in diet and lifestyle [2].

The International Diabetes Federation (IDF) indicates that the proportion of adults living with diabetes is increasing in Saudi Arabia, doubling from approximately 23.1% in 2024 to more than 25.4% in 2025, posing an increasing public health burden [3]. It is projected that one of every three adults in the country may have diabetes by 2050 [4]. The rising epidemic is primarily due to multiple factors, such as a high-calorie diet, lack of exercise, and an aging population [5]. In addition, urbanization and economic growth have led to a rising incidence of obesity, which is strongly correlated with the development of T2D [6]. All these issues highlight the urgent need for effective public health interventions [7].

As T2D becomes more prevalent, it is essential to understand the determinants of its incidence and develop predictive models to estimate the number of T2D cases. The study seeks to fill the gaps by identifying the most significant KPIs associated with T2D and developing a reliable model to estimate the monthly number of new T2D cases using local data. The findings of this research will equip healthcare practitioners and policymakers in Saudi Arabia to develop effective public health initiatives and interventions to address the mounting problem of T2D. Various statistical methods, including Poisson Regression (PR), Negative Binomial Regression (NBR), Poisson Inverse Gaussian Regression (PIGR), and Bell Regression (BR), have been employed to predict and identify the KPIs that significantly impact the number of T2D cases.

## 2 Literature review

The incidence of T2D is increasing worldwide, representing more than ninety percent of all cases of diabetes globally [8]. The incidence rates of T2D vary across different populations worldwide. This variation is likely attributed to differences in insulin sensitivity, which is influenced by the interaction of environmental and genetic factors among various ethnic and demographic groups [9]. Saudi Arabia is not immune to this global epidemic, witnessing a significant rise in diabetes cases [10]. It has been reported that the prevalence of diabetes among the Saudi population has reached concerning levels [11]. Despite this growing burden, research on T2D in Saudi Arabia remains limited, particularly on predicting the number of T2D cases and conducting comprehensive risk factor analyses.

Models used to perform regression analysis of count data are nonlinear, due to the discreteness and non-negativity properties of the counts. The Poisson regression (PR) is commonly used for analyzing such data [12,13]. However, PR relies on the premise that the mean and variance of the count measure are equal, a concept known as equidispersion. In real populations, empirical data often exhibit overdispersion (the variance exceeds the mean) [14]. Ignoring the existence of overdispersion in data may lead to underestimated standard errors, inflated test statistics, and spuriously high significance levels [13]. Due to this limitation, researchers have explored mainly alternative modeling methods, such as negative binomial regression (NBR), which handles overdispersed counts better [13]. The respective models are widely applied in various fields to analyze count outcomes, such as forecasting accident rates [15], measuring the frequencies of medical consultations [16], and analyzing epidemiological or public health data on the incidence or prevalence of diseases [17].

Research on assessing the efficacy of these models has yielded mixed results, depending on the specific framework and properties of the dataset. In an extensive international study, NBR was used to quantify the incidence of T2D among 41,600 children and adolescents from various regions. The analysis revealed that body mass index (BMI), age, sex, and geographic location were significant predictors of T2D. The NBR model proved effective in addressing overdispersion, and the results indicated that China, India, and America are the most important contributors to the global burden of diabetes. A notable finding was that Western Pacific nations and higher-middle-income countries were responsible for 41% of the new global cases, highlighting the strong association of higher BMI and rising incidence rates [18]. An NBR model was used to analyze trends in type 1 diabetes and T2D incidence rates in the US. The results showed an annual increase of 1.9% in type 1 diabetes and 4.8% in T2D. The incidence was significantly increased among ethnic minority groups of American Indians, African Americans, and Hispanics. The authors used the incidence rate ratios to further investigate the impact of obesity, physical inactivity, and socio-economic differences on diabetes incidence [19].

In China, a multiple Poisson regression (MPR) model was employed to analyze data from 879,769 patients with T2D. The study used variables such as age, sex, and residence area (urban or rural). The results showed a significant increase in the prevalence of diabetes, especially in males, young populations, and those residing in rural areas. The results revealed a clear increasing trend in incidence rates over 10 years, highlighting the growing burden of diabetes [20]. Researchers in Bangladesh employed the MPR model to examine the age-standardized incidence rates of both prediabetes and diabetes in a sample of 11,952 adults. The study reported an overall prevalence rate of 9.2%. A higher prevalence was observed in females (9.6%) compared to males (8.8%). The major determinants associated with the prevalence of diabetes included age, BMI, hypertension, wealth quintile, and geographical area. The study also showed that prevalence rates were very high among the elderly, especially those aged 70 years and older, and obesity and hypertension were significant risk factors [21]. In the United States, a PR model was used to predict the incidence rates of early-onset T2D between 2001 and 2020. The study showed a marked increase in incidence among young adults, particularly in socioeconomically disadvantaged groups. BMI was identified as a significant contributing factor [22].

Local studies in Saudi Arabia have significantly contributed to predicting incidence rates and disease prevalence. A systematic review and meta-analysis of cross-sectional studies between 2016 and 2022 were performed to analyze the prevalence rates of T2D and associated risk factors among the general adult population. The study pooled data from 10 studies involving 8,457 participants. Variables assessed included age, sex, obesity, and smoking. The findings showed that the prevalence rate of T2D was 28%. A significantly higher risk was observed in individuals over the age of 40 (OR = 1.74, 95% CI = 1.34–2.27) [11]. Another study investigated the prevalence of T2D and its associated risk factors in a semi-urban area. The study included 353 participants and assessed variables such as age, high-density lipoprotein level (HDL), sex, total cholesterol level (TC), lifestyle factors, triglyceride level (TG), and BMI. The findings indicated that 34.6% of individuals were diagnosed with T2D. Obesity and elevated TG were identified as major contributors (p < 0.001 and p < 0.004, respectively) [23].

Additionally, an extensive analysis of data covering 24,012 households was conducted, focusing on age, sex, and geographic differentials as variables. The use of prevalence mapping, in conjunction with descriptive statistics, facilitated the identification of the national and regional prevalence of diabetes. The results revealed a higher prevalence among males (10.3%) than females (9.9%). The highest prevalence of diabetes was observed in individuals aged 60 years and above (49.2%), followed by those aged 45–64 years (38.9%), while the lowest incidence was recorded in the younger age group under 40 years (15.3%) [24]. Another study confirmed that the prevalence of T2D had increased considerably and was the highest among men and individuals aged > 40 years [25].

Despite the alarming increase in diabetes prevalence in Saudi Arabia, no attempts have been made to develop predictive models to estimate the future number of T2D cases in the country. Previous studies conducted in Saudi Arabia [11,23–25] have methodological limitations, including reliance on cross-sectional designs with small and non-representative samples, as well as the examination of only a limited number of variables, which restricts the generalizability of their findings. In addition, national estimates reported by organizations such as the World Health Organization (WHO) and the International Diabetes Federation (IDF) relied heavily on statistical projections rather than primary local data, raising concerns about their accuracy in the Saudi population context. To the best of our knowledge, this is the first study to apply multiple count regression models using a large sample of local data, along with an extended number of factors recommended in national and international existing literature, to develop a reliable predictive framework tailored specifically to the Saudi population. Several past studies have employed PR in various contexts, including China, Bangladesh, and the United States, to demonstrate its effectiveness in estimating diabetes incidence [20–22]. Other studies have also highlighted that the PR model is an effective method for forecasting case numbers of various diseases [26,27].

This study aims to fill the gap in the existing literature by utilizing local data specifically collected for this research to model the monthly number of new cases of T2D in Saudi Arabia using Poisson Regression (PR), Negative Binomial Regression (NBR), Poisson Inverse Gaussian Regression (PIGR), and Bell Regression (BR). This study also focuses on comparing the performance of the full models, which include the most recommended variables, with the reduced models, which focus on the most significant factors identified by full models, to provide valuable insights into the impact of the most essential KPIs on the monthly number of new cases of T2D. Through a better understanding of the number of new T2D cases and their corresponding most significant KPIs, medical practitioners can develop strategies to enhance public health prevention and treatment in Saudi Arabia.

## 3 Materials and methodology

### 3.1 Data collection

The medical records of 4,526 patients aged 20 years or older who were diagnosed with T2D at King Abdulaziz University Hospital (KAUH) in Jeddah, Saudi Arabia, were used to extract de-identified data for the analysis. Ethical approval was provided by the Human Research Ethics Committee at RMIT University in Australia and the Research Ethics Committee at KAUH. Ethical clearance and access to the de-identified data were granted on 19/11/2023. The dataset included patients diagnosed with T2D between 01/01/2018 and 31/12/2022. The Research Ethics Committee at KAUH waived the need for informed consent, as the study used existing de-identified data, which does not require written or verbal consent for this type of research. To ensure data quality, the following filtering steps were taken: first, variables with over 90% missing data were excluded; then, variables with very low variance (i.e., where at least 90% of the patients shared the same value) were removed from the analysis. As a result, the number of variables was reduced from 35 to 19. After selecting the final 19 variables, we excluded 1,526 records with substantial missing information that could not be reliably imputed, along with 2,000 non-diabetic records, from the original dataset of (N = 4,526) observations. As a result, the dataset used for the analysis consisted of (n = 1,000) diabetic patients. Subsequently, the missing values in the remaining records were handled using mean imputation for numerical variables and mode imputation for categorical variables.

### 3.2 Dependent variable and independent variables

The sum of the monthly diagnoses of T2D from 2018 to 2022 served as the dependent variable in this study. The KPIs included in the analysis consist of categorical and numerical variables. Gender, nationality, smoking habits, exercise habits, eating habits, presence of hypertension (yes/no), employment status (employed/unemployed), and marital status (married/unmarried) represent the categorical variables. The numerical variables included age, glycated hemoglobin (HbA1c), high-density lipoprotein (HDL), diastolic blood pressure (DBP), total cholesterol (TC), systolic blood pressure (SBP), vitamin D levels, white blood cell count (WBC), triglycerides (TG), ferritin and body mass index (BMI), as illustrated in Table 1. The participants classified as patients in this study presented HbA1c values above 6.5%.

**Table 1:** Summary of case count and percentage of predictors associated with T2D.

Variables	n (%)	Variables	n (%)
**Year**		**Occupation**	
2018	77(7.7%)	Employed	288(28.8%)
2019	49(4.9%)	Not Employed	712(71.2%)
2020	37(3.7%)	**Smoking**	
2021	396(39.6%)	Yes	869(86.9%)
2022	441(44.1%)	No	131(13.1%)
**Gender**		**Physical Activity**	
Female	446(44.6%)	Yes	871(87.1%)
Male	554(55.4%)	No	129(12.9%)
**BMI**		**Type of Food**	
Underweight	22(2.2%)	Heathy	351(35.1%)
Normal weight	184(18.4%)	Non healthy	649(64.9%)
Overweight	337(33.7%)	**Hypertension**	
Obese	457(45.7%)	Yes	782(78.2%)
**Marital Status**		No	218(21.8%)
Married	818(81.8%)	**Age**	
Not Married	182(18.2%)	20-39	89(8.9%)
**Nationality**		40-59	459(45.9%)
Saudi Arabia	639(63.9%)	60-79	216(21.6%)
Not Saudi Arabia	361(36.1%)	80 ≤	236(23.6%)
**TC**		**HDL**	
Normal	223(22.3%)	Low	585(58.5%)
Moderately	206(20.6%)	Good	224(22.4%)
High	571(57.1%)	High	191(19.1%)
**TG**		**Vitamin- D**	
Normal	198(19.8%)	Deficient	478(47.8%)
Moderately	206(20.6%)	Insufficient	231(23.1%)
High	596(59.6%)	Sufficient	291(29.1%)
**WBC**		**Ferritin**	
Low	46(4.6%)	Deficient	581(58.1%)
Good	458(45.8%)	Insufficient	230(23%)
High	496(49.6%)	Sufficient	189(18.9%)
**SBP**		**DBP**	
Low	166(16.6%)	Low	668(66.8%)
Good	381(38.1%)	Good	181(18.1%)
High	453(45.3%)	High	151(15.1%)

HDL: High-Density Lipoprotein level, BMI: Body Mass Index, TC: Total Cholesterol Level, SBP: Systolic Blood Pressure, DBP: Diastolic Blood Pressure, TG: Triglycerides Level, WBC: White Blood Cells.

Table 1 presents the statistical distribution of T2D cases in relation to demographic, lifestyle, and lipid profile variables. In 2022, the percentage of T2D cases was the highest at 44.1%, followed by 39.6% in 2021, indicating a notable upward trend in the prevalence of T2D cases over recent years. The gender distribution analysis revealed a slightly higher prevalence among males (55.4%) compared to females (44.6%). The high incidence was among the 40–59 years cohort at 45.9% followed by the 80 years and above group at 23.6%, the 60–79 years group at 21.6%, and the 20–39 years group at 8.9%. The findings indicate a high risk of T2D among the older cohort, particularly those aged 40 years and above. According to BMI screening, most participants were obese (45.7%) or overweight (33.8%), further confirming the previously reported research on the relationship between overweight and diabetes.

We noted that 82% of the participants were married, and a high incidence of T2D was observed among those with high physical activity (87.1%), poor diet (64.9%), smoking (86.9%), and unemployment (71.2%). For analysis purposes, the variable physical activity was recoded into two categories: ‘Yes’ (patients reporting any level of mobility or physical activity, such as ‘free’, ‘low activity’, or ‘yes’) and ‘No’ (patients with restricted mobility or severe impairment, such as ‘bedridden’, ‘amputation’, or ‘no’). Similarly, the variable type of food was recoded into two categories: ‘Healthy’ (patients on specific medically advised diets, e.g., diabetic, renal, low salt, cholesterol-restricted, or heart-healthy diets) and ‘non-healthy’ (patients reporting a regular diet, unspecified diets, or no dietary modification). The analysis revealed hypertension in 78.2% of the population, elevated systolic blood pressure in 45.3%, and low diastolic blood pressure in 66.8%, indicating possible dysfunction in cardiovascular regulation.

There were abnormalities in the lipid profile, with 57.1% having high TC, 59.6% having elevated TG, and 58.5% having low HDL cholesterol, all strongly correlated with insulin resistance. 47.8% showed vitamin D deficiency, and 58.1% showed low ferritin levels, implying an inflammatory state, nutritional insufficiency, or both. In addition, high leukocyte counts were noted in nearly half of the patients, thereby augmenting the inflammatory load commonly seen in T2D.

## 4 Model development

Poisson Regression (PR), Negative Binomial Regression (NBR), Poisson Inverse Gaussian Regression (PIGR), and Bell Regression (BR) were employed to predict the monthly number of new T2D cases and to identify the key performance indicators (KPIs) significantly associated with the number of T2D cases in Saudi Arabia. The analysis was conducted using the statistical software R. All code for data analysis associated with the current submission is available at https://github.com/fatenalhussains/Count_Regression_T2D_Saudi/blob/main/T2D_Count_Regression_Code.R.

### 4.1 Count regression models

Count data is a measure of the frequency with which an event occurs within a particular time interval. It is widely available in highway safety, traffic accident analysis, biostatistics, and demographics [28,29]. The PR model is often used for statistical modeling of count data [30]. However, the common problem that may result in poor fitting in the PR model is overdispersion (where the variance exceeds the mean). For data with overdispersion, the NBR model is generally preferred [31]. The PIGR model is also recommended for analysis in the presence of overdispersion, as the conditional mean of a PIGR is μ(x), and the variance is given by $\mu (x)+\;\tau \mu {\left(x\right)}^{\text{2}}$ with τ>0, which implies that the variance is always at least as large as the mean [32]. Last, the BR model is efficient in reducing overdispersion and obviating the need for an additional dispersion parameter [33].

#### 4.2.1 Poisson Regression model (PR).

Poisson Regression (PR) is a statistical modeling technique used to analyze count data, where the dependent variable represents the number of occurrences of a specific event within a given time period. It is commonly used when the data follows a Poisson distribution, denoted as:

$Y_{i}\sim Poisson\left(\lambda_{i}\right)$

Where $Y_{i}$ is the response variable that counts the number of cases, and $\lambda_{i}$ is the rate parameter representing the expected number of occurrences. This distribution assumes that the mean of the distribution equals its variance [34].

In the context of PR, the relationship between the independent variables (predictors) and the dependent variable (count) is modeled using a log-linear function. The model can be expressed mathematically within the framework of generalized linear models (GLM), as shown in equation (1) [34,35]:


$$\begin{array}{c} Log\left(\lambda_{i}\right)=x_{i}^{\prime }\beta =\beta_{\text{0}}+\sum_{j=\text{1}}^{p}\beta_{j}x_{ij};i=\text{1},\text{2},\ldots ,n \end{array}$$ (1)

Where the observed values $Y_{i}\sim Poisson\left(\lambda_{i}\right)$, $\lambda_{i}$ denote the expected count for the i−th observation, with a vector of predictors $x_{i}^{\prime }=(\text{1},x_{i\text{1}},\;x_{i\text{2}},\ldots ,x_{ip})$ and $\beta ={\left(\beta_{\text{0}},\beta_{\text{1}},\;\ldots ,\beta_{p}\right)}^{\prime }$ representing the vector of coefficients. The estimation of the coefficients is achieved by maximizing the log-likelihood function, where the log-likelihood function for β [36] is given by:


$$\begin{array}{c} \text{𝓁}(\beta ;y)=\sum_{i=\text{1}}^{n}\left[y_{i}x_{i}^{\prime }\beta -e^{x_{i}^{\prime }\beta }-\text{log}(y_{i}!)\right]. \end{array}$$

#### 4.2.2 Negative Binomial Regression model (NBR).

The Negative Binomial Regression (NBR) is a statistical technique designed to model the relationship between a dependent variable and one or more independent variables in the context of count data. It serves as an extension of the PR model, particularly addressing the issue of overdispersion [37]. This model is based on the negative binomial distribution, which provides greater flexibility in accommodating variability in count data [35]. The general equation for the NBR model is expressed as shown in equation (1).

The NBR model is derived from the Poisson-Gamma mixture and is commonly used to address the issue of overdispersion in count data [35]. The expected value and variance of $Y_{i}$ are given by $\text{E}\;(Y_{i}=\;\lambda_{i}$ and $\text{Var}\;(Y_{i}=\;\lambda_{i}\;+\;\nu \lambda_{i}^{\text{2}}$, respectively, for i=1,…,n.

This formulation allows the NBR model to effectively manage datasets where the variance is significantly larger than the mean, making it a robust alternative to PR in such scenarios [38]. For this regression model, $\beta ={\left(\beta_{\text{0}},\beta_{\text{1}},\;\ldots ,\beta_{p}\right)}^{\prime }$ denotes the vector of regression coefficients, and $x_{i}^{\prime }=(\text{1},x_{i\text{1}},\;x_{i\text{2}},\ldots ,x_{ip})$ represents the vector of predictors for the i−th observation. The model parameters are estimated via maximum likelihood estimation (MLE), where the log-likelihood function for the parameters (β,v) is given by [35]:

$\begin{array}{c} \text{𝓁}(\beta ,v)=\sum_{i=\text{1}}^{n}\left[\text{ln}\Gamma \left(y_{i}+v^{-\text{1}}\right)-ln\;\Gamma \left(v^{-\text{1}}\right)-lny_{i}!-y_{i}ln\left(\text{1}+ve^{x_{i}^{\prime }\beta }\right)-v^{-\text{1}}\text{ln}\left(\text{1}+ve^{x_{i}^{\prime }\beta }\right)+y_{i}ln\;v+y_{i}x_{i}^{\prime }\beta \right]. \end{array}$

#### 4.2.3 Poisson Inverse Gaussian Regression model (PIGR).

The Poisson Inverse Gaussian regression (PIGR) model is an advanced statistical method developed for count data to overcome the limitation of overdispersion, thus extending the properties of the traditional PR and NBR models [39]. Through the combination of the inverse Gaussian distribution and the Poisson distribution, the PIGR model enhances the analytical flexibility required to analyze data sets with significant variability or influenced by the presence of outliers [40]. The model is particularly beneficial in the health sciences discipline, where the data could include very rare or extreme events, leading to the presence of unobserved heterogeneity [41]. The PIGR model follows the same log-linear form presented in equation (1). This distribution is a mixture of a Poisson distribution and an Inverse Gaussian distribution. Specifically, conditional on V, the response variable follows [32]:


$$Y_{i}\sim Poisson\left(V\lambda_{i}\right),\;where\;\;V\sim IG(\text{1},\;\frac{\text{1}}{\tau })$$

Here, V is assumed to follow an Inverse Gaussian distribution with mean equal to 1 and dispersion parameter $\frac{\text{1}}{\tau }$.

This formulation enables the PIGR model to effectively account for the excess variance often observed in complex datasets, making it a robust tool for statistical analysis in scenarios where conventional models may be inadequate [40]. The probability generation function of PIGR (μ,τ), is shown in equation (2) [32]:


$$\begin{array}{c} P(z)=\;\sum_{y=\text{0}}^{\infty }p(Y=y)z^{y}=e^{\left(\tau^{-\text{1}}\left[\text{1}-{\left\{\text{1}-\text{2}\tau \mu (z-\text{1})\right\}}^{\frac{\text{1}}{\text{2}}}\right]\right)}, \end{array}$$ (2)

In this model, $\beta ={\left(\beta_{\text{0}},\beta_{\text{1}},\;\ldots ,\beta_{p}\right)}^{\prime }$ denotes the vector of coefficients and $x_{i}^{\prime }=(\text{1},x_{i\text{1}},\;x_{i\text{2}},\ldots ,x_{ip})$ represents the vector of predictors for the i−th observation. The model parameters are estimated via MLE, where the log-likelihood function from a random sample of observations ($y_{i},x_{i}^{\prime })$ is 𝓁$\begin{array}{c} (\beta ;\tau )\;=\;\sum {\mathrm{\backslash nolimits}}_{i=\text{1}}^{n}\text{log}\;p_{i}\left(y_{i}\right) \end{array}$, where $p_{i}\left(y_{i}\right)$ stands for $\text{P}\left(Y=y_{i}|x_{i}^{\prime };\beta ,\tau \right)$. For convenience, we write $\mu_{i}=\mu (x_{i}^{\prime };\beta )$,


$$t_{i}(y)=(y+\text{1})\frac{p_{i}(y+\text{1})}{p_{i}(y)},\;y=\text{0},\text{1},\text{2},\ldots$$ (3)

By applying equations (2) and (3), the log-likelihood function 𝓁(β;τ) can be expressed as follows:


$$(\beta ;\tau )=\sum_{i=\text{1}}^{n}\left(\text{log}\left(\frac{\text{1}}{y_{i}!}\right)+\text{log}\;p_{i}(\text{0})+I(y_{i}>\text{0})\sum_{j=\text{0}}^{y_{i}-\text{1}}\text{log}\;t_{i}(j)\right)$$

#### 4.2.4 Bell Regression model (BR).

The Bell Regression (BR) is a regression model based on the Bell distribution. It offers simplicity in calculations and can serve as a valuable alternative to PR and NBR models [33] used for count response variables. The probability mass function of this distribution is presented in equation (4) [33]:


$$P(Y=y)=\frac{\lambda^{y}e^{-e^{\lambda \;}+\text{1}}B_{y}}{y!}\;,\;\;\;\;y=\text{0},\text{1},\text{2},\ldots$$ (4)

where λ>0 and $B_{y}=\;\frac{\text{1}}{e}\sum_{d=\text{0}}^{\infty }\frac{d^{y}}{d!}$ denotes the bell numbers. The following statistical properties characterize the Bell distribution:


$$E(Y)=\lambda e^{\lambda }$$

$$Var(Y)=\;\lambda e^{\lambda }(\text{1}+\lambda )$$

Assuming $\psi =\lambda e^{\lambda }$ and $\lambda =W_{\text{o}}(\psi )$ where $W_{o}(.)$ is the Lambert function. Then equation (4) can be written in the new parameterization as:


$$P(Y=y)=\;e^{\left(\text{1}-e^{W_{\text{o}}(\psi )}\right)}\frac{{W_{\text{o}}(\psi )}^{y}\;B_{y}}{y!}$$

In the BR model, the parameter $\psi_{i}$ is defined as $\psi_{i}=\;e^{{(x}_{i}^{\prime }\beta )}e^{e^{\left(x_{i}^{\prime }\beta \right)}}$, and the response variable follows $y_{i}=Bell\;(W_{o}\left(\psi_{i}\right))$, where $\beta ={\left(\beta_{\text{0}},\beta_{\text{1}},\;\ldots ,\beta_{p}\right)}^{\prime }$ represents the model parameters, and $x_{i}^{\prime }=(\text{1},x_{i\text{1}},\;x_{i\text{2}},\ldots ,x_{ip})$ denotes the set of predictors for observation i−th. These parameters are estimated using the MLE method, where the log-likelihood function [33] is given by:


$$\begin{array}{c} \text{𝓁}(\beta ,\lambda )=\sum_{i=\text{1}}^{n}{y_{i}loge}^{x_{i}^{\prime }\beta }e^{e^{x_{i}^{\prime }\beta }}+\sum_{i=\text{1}}^{n}(\text{1}-e^{e^{x_{i}^{\prime }\beta e^{e^{x_{i}^{\prime }\beta }}}})+logB_{y}-log\left(\prod_{i=\text{1}}^{n}y_{i}!\right). \end{array}$$

## 5 Testing for variable dispersion

In this section, we calculate the Poisson dispersion index (DI) to assess the distribution of data, including dispersion, underdispersion, and overdispersion, in the number of cases using the variance-to-mean ratio for the random variable Y. The dispersion index is defined by [42].


$$Dispersion\;Index\;(DI)=\frac{Var(Y)}{E(Y)}$$

If the DI is greater than 1, it indicates the presence of overdispersion; if the DI is less than 1, it suggests that the data is underdispersed [43]. This approach helps the researchers to identify the appropriate statistical model for analyzing count data while reducing the impact of overdispersion or underdispersion on the accuracy of model predictions [44].

## 6 Performance evaluation measures

A set of standard evaluation metrics was employed to assess the performance of PR, NBR, PIGR, and BR, in predicting the monthly number of diabetes cases. These metrics include the coefficient of determination (R²), root mean squared error (RMSE), mean absolute error (MAE), 10-fold cross-validation (CV-10), Akaike information criterion (AIC), and Bayesian information criterion (BIC).

The R² value indicates how well the model explains the variability in the outcome variable. A higher value reflects a better fit. The RMSE and MAE measure the average prediction error, with lower values indicating better model accuracy [45,46]. In addition, the CV-10 provides an estimate of the model’s generalizability by measuring performance consistency across ten data folds [47]. AIC and BIC are information-based criteria used to assess the model fit, where lower values indicate better model performance, striking an optimal balance between goodness of fit and model complexity [48]. The AIC and BIC are defined by:


AIC or BIC= −2 log(l)+τk

where k is the number of model parameters and l is the maximized value of the likelihood function. The AIC uses  τ=2, whereas the BIC uses  τ=log(n), where n represents the total number of observations used in the calculation of likelihood function.

Additionally, the Likelihood Ratio Test (LRT) can be used to statistically compare nested models by evaluating the difference between their log-likelihoods. The LR test is instrumental when testing whether a reduced model fits the data as well as the full model. The test statistic is calculated as:


$$LR=-\text{2}\left\{L_{reduce}-L_{full}\right\}$$

where $L_{reduce}$ and $L_{full}$ represent the maximized log-likelihoods of the reduced and full models, respectively, under the null hypothesis that the reduced model fits the data as well as the full model. The LR statistic asymptotically follows a chi-square distribution with degrees of freedom equal to the difference in the number of parameters between the models. A non-significant p-value suggests that the reduced model describes the data as well as the full model, favoring model simplicity without significant loss of information [36]. These evaluation metrics provide a comprehensive assessment of model performance to determine the most effective approach for predicting the number of T2D cases.

## 7 Results analysis

In this study, we transformed individual-level data into monthly aggregated values to analyze trends and patterns at the population level. For continuous numerical variables, such as age or BMI, we calculated the monthly averages. This involved computing the mean value of each variable for all patients diagnosed in that particular month. For binary categorical variables, such as gender or smoking status, we determined the monthly proportion of patients in the positive class, defined as those with a value of 1. This proportion represents the percentage of diagnosed patients each month who exhibit the characteristic of interest.

Table 2 displays the result of the overdispersion test for the response variable. The null hypothesis ($H_{\text{0}}$) assumes the actual dispersion parameter is one, reflecting the absence of overdispersion. The alternative hypothesis ($H_{\text{1}}$) suggests that the actual dispersion parameter is greater than one, reflecting the presence of overdispersion. The test statistic (z = 2.879) yields a p-value of 0.001, leading to rejection of the null hypothesis.

**Table 2:** Results of the overdispersion test of the dependent variable (y).

Dispersion Index value	z	p-value
7.435	2.879	0.001*

* Accept the alternative hypothesis ( $H_{\text{1}}$ ): true dispersion is greater than one.

**Fig 1:**
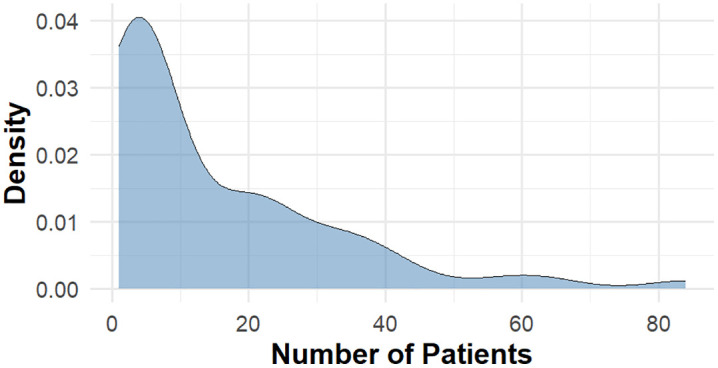
Smoothed distribution of the number of T2D cases using kernel density estimation (KDE).

Fig 1 illustrates that the variance of the count variable significantly exceeds the mean. The estimated value for the dispersion parameter also confirms the presence of overdispersion. Such a violation indicates that the PR model is not suitable for the dataset at hand, as it might lead to underestimated standard error and inflated type I error rates (false positives). Alternative count data models, such as NBR, PIGR, and BR, should be used to handle overdispersion.

To predict the number of T2D cases each month in Saudi Arabia, predictive models PR, NBR, PIGR, and BR are used. In these models, the monthly number of T2D cases served as the dependent variable (y), while all the variables listed in Table 1 were used as independent variables (x’s). Table 3 presents the four full count regression models together with their performance evaluation metrics.

**Table 3:** Evaluation measures of the full PR, full NBR, full PIGR, and full BR models.

Model	$R^{2}$	RMSE	MAE	AIC	BIC	CV-10
Full PR	0.53	2.23	1.82	951.83	962	5.74
Full NBR	0.88	0.93	0.69	873.23	880	1.21
Full PIGR	0.68	1.67	1.32	901.02	910	3.29
Full BR	0.73	1.49	1.15	890.11	899	2.25

Table 3 illustrates that the full NBR model outperforms all other models in terms of predictive performance and model fit. It achieves the highest R² value (0.88), indicating superior explanatory power, along with the lowest RMSE (0.93), MAE (0.69), and CV-10 (1.21), reflecting greater prediction accuracy. Additionally, the full NBR model has the lowest AIC (873.23) and BIC (880), confirming its statistical adequacy and lower complexity compared to the other models. Furthermore, the full BR model also demonstrates better performance, with a relatively high R² value (0.73), moderate error rates (RMSE = 1.49, MAE = 1.15, CV-10 = 2.25), and lower AIC (890.11) and BIC (899) compared to the full PR and full PIGR models, highlighting it as a reliable and competitive alternative. In contrast, the full PR model shows the weakest performance across all metrics, with the lowest R² (0.53) and the highest error values and information criteria. Although the full PIGR model performs reasonably well compared to the full PR model, it still lags behind the full NBR and BR models in terms of predictive accuracy and model fit.

**Fig 2:**
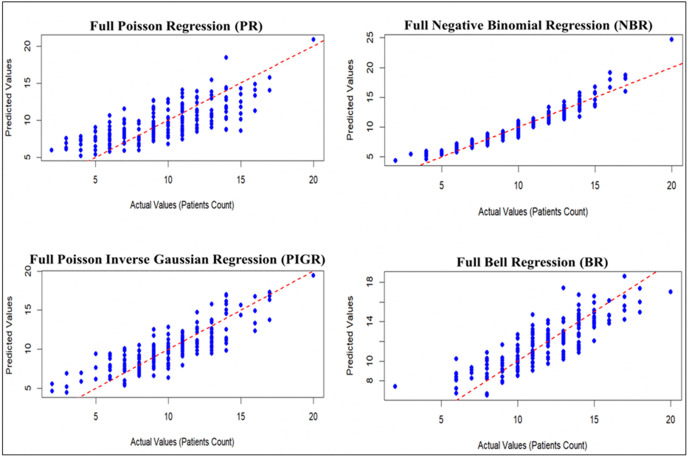
Plot of actual against predicted values for the full PR, NBR, PIGR, and BR models.

Fig 2 compares the predictive performance of the four regression models (full PR, full NBR, full PIGR, and full BR) based on actual patient counts. The NBR model demonstrates superior performance, achieving the closest agreement between predicted and actual values.

**Fig 3:**
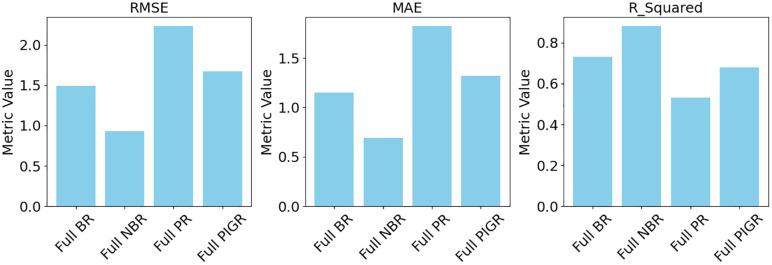
Model comparison across evaluation metrics.

**Fig 4:**
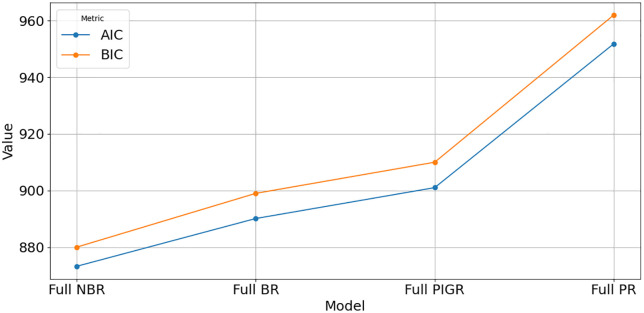
Comparison of AIC and BIC across full models.

Figs 3 and 4 illustrate that the full NBR and full BR models offer better predictive accuracy and model adequacy for estimating T2D cases, as they exhibit the lowest error metrics (RMSE and MAE) and the highest model fit indicators (R², AIC, and BIC), compared to the full PR and PIGR models.

Table 4 presents an overview of the coefficients and p-values obtained from the full models, highlighting the significance of each variable in predicting the monthly incidence of new T2D cases. The results indicate that marital status, occupation, cigarette smoking, age, BMI, DBP, HDL, and TC were statistically significant predictors (p < 0.05) in the PR model. Most of the variables had a positive relationship with the incidence rate of T2D. On the other hand, HDL had an inverse relationship, indicating that high levels of HDL are associated with a decrease in the incidence of T2D. Gender, physical exercise, nationality, hypertension, diet, HbA1c, WBC, SBP, TG, ferritin, and vitamin D were not statistically significant predictors (p ≥ 0.05).

**Table 4:** Estimation results of count data regression models.

	Full PR		Full NBR		Full PIGR		Full BR	
Variable	Coeff	p-value	Coeff	p-value	Coeff	p-value	Coeff	p-value
Constant	0.697	0.000 ***	3.090	0.000 ***	2.666	0.022*	0.171	0.036*
Gender	−0.091	0.178	0.005	0.959	−0.333	0.411	0.008	0.962
Marital Status	0.411	0.021*	0.523	0.000 ***	0.367	0.002**	0.292	0.018*
Occupation	0.242	0.033*	0.261	0.023*	0.238	0.031*	−0.052	0.077
Physical activity	0.369	0.301	0.214	0.041*	0.292	0.004**	0.133	0.525
Smoking	0.162	0.011*	0.042	0.733	−0.432	0.258	0.327	0.011*
Nationality	0.148	0.299	−0.532	0.000 ***	0.322	0.267	0.450	0.004**
Hypertension	0.673	0.607	0.499	0.000 ***	−0.138	0.412	0.406	0.031*
Type of Food	−0.182	0.205	−0.409	0.002**	−0.392	0.213	0.018	0.093
Age	0.394	0.041*	0.454	0.000 ***	0.092	0.000 ***	0.495	0.000***
BMI	0.273	0.009**	0.147	0.002**	0.030	0.041*	0.263	0.023*
HbA1c	0.149	0.453	0.151	0.192	0.195	0.202	0.386	0.044*
SBP	−0.346	0.842	−0.225	0.092	−0.055	0.009**	−0.345	0.094
WBC	0.464	0.719	−0.410	0.028*	−0.112	0.000***	0.361	0.001**
DBP	0.535	0.046*	0.147	0.256	0.148	0.679	−0.182	0.386
HDL	−0.070	0.042*	− 1.663	0.000 ***	−0.261	0.000***	−0.087	0.002**
TC	0.118	0.001**	0.462	0.001 **	0.201	0.000 ***	0.453	0.044*
TG	0.130	0.055	0.483	0.000 ***	0.213	0.288	0.137	0.066
Ferritin	0.285	0.832	−0.329	0.013*	0.069	0.031*	0.171	0.434
Vitamin D	0.135	0.141	−0.711	0.000 ***	0.235	0.364	0.508	0.000***

*** Significant at p-value <0.001.

** Significant at p-value <0.01.

* Significant at p-value <0.05.

A detailed analysis of the overall NBR model revealed that variables positively correlated with the predicted incidence of T2D cases (p < 0.05) included marital status, occupation, physical activity, hypertension, age, BMI, TC, and TG. The following six variables showed negative association with the incidence of T2D cases: nationality, diet, WBC, HDL, ferritin, and vitamin D. Gender, smoking, HbA1c, SBP, and DBP were not statistically significant (p ≥ 0.05).

The analysis of the full PIGR model revealed that marital status, occupation, physical activity, age, BMI, WBC, SBP, HDL, TC, and ferritin were significant variables. Gender, smoking, nationality, hypertension, type of food, HbA1c, DBP, TG, and vitamin D were not statistically significant (p ≥ 0.05).

In the full BR model, marital status, smoking, nationality, hypertension, age, BMI, HbA1c, WBC, HDL, TC, and vitamin D have p-values less than 0.05, indicating their significant contribution to the model. Gender, occupation, physical activity, dietary type, SBP, DBP, TG, and ferritin were not statistically significant (p ≥ 0.05).

## 8 Significant variables associated with predicting the number of T2D cases in each model

Significant factors predicting the expected number of T2D cases, identified through the full PR, NBR, PIGR, and BR models, are marked with an asterisk (*) in Table 5. The results show that marital status, age, BMI, HDL, and TC were statistically significant across all four models. Additionally, WBC and occupation were significant in at least three models, suggesting a relatively consistent influence on estimating the number of new T2D cases. This statistical consistency across different modeling approaches enhances their importance as reliable predictors, reinforcing their role in explaining variation in the expected number of diabetes cases. Gender did not show statistical significance in any of the models.

**Table 5:** Significant variables in full PR, NBR, PIGR, and BR models.

Variable	Full PR	Full NBR	Full PIGR	Full BR
Gender				
Marital Status	*	*	*	*
Occupation	*	*	*	
Physical activity		*	*	
Smoking	*			*
Nationality		*		*
Hypertension		*		*
Type of Food		*		
Age	*	*	*	*
BMI	*	*	*	*
HbA1c				*
SBP			*	
WBC		*	*	*
DBP	*			
HDL	*	*	*	*
TC	*	*	*	*
TG	*	*		
Ferritin		*	*	
Vitamin D		*		*

(*) Indicates that the variable has a significant impact on the model.

## 9 Reduced PR, NBR, PIGR, and BR models based on significant variables

In this section, reduced PR, NBR, PIGR, and BR models were constructed using the variables that were significant in all the full models, namely marital status, BMI, age, HDL, and TC. This approach aims to evaluate the efficiency of models using fewer variables and to compare their performance with that of the full models in terms of accuracy and predictive performance in estimating the number of T2D cases. The results presented in Table 6 show that the reduced NBR model outperformed the PR, PIGR, and BR models, achieving the highest R² value (0.84), along with the lowest RMSE, MAE, and CV-10 (1.1, 0.86, and 1.37), AIC (899), and BIC (910). These findings highlight that the reduced models, which include only five significant predictors, predict the number of new T2D cases with almost the same accuracy as the full model (R² of 0.84 compared with R² = 0.88 in the full model).

**Table 6:** Performance metrics of reduced PR, NBR, PIGR, and BR models using the most significant predictors across all models.

Model	$R^{2}$	RMSE	MAE	AIC	BIC	CV-10
Reduced PR	0.49	2.33	1.84	958.07	970	6.32
Reduced NBR	0.84	1.10	0.86	899	910	1.37
Reduced PIGR	0.63	2.24	1.82	910.56	922	4.81
Reduced BR	0.71	1.59	1.24	903.22	912	2.36

In the context of comparing the full and reduced models in this study, a noticeable variation appears in the values of the AIC that carries considerable statistical significance and reflects differences in the quality of model fit. The AIC value for the full NBR model was approximately 873.23, whereas it increased to 899 in the reduced version of the same model, representing a difference of about 25.77 units. This difference is statistically significant, supporting the superiority of the full model in terms of estimation quality and statistical goodness of fit.

However, the selection of the reduced model was not arbitrary or merely driven by a desire to reduce the number of variables. Instead, it followed a methodological framework aimed at achieving a balance between model accuracy and simplicity. Despite the relative increase in the AIC value, the reduced model maintained a strong level of explanatory power. The coefficient of determination $R^{\text{2}}$ for the reduced NBR model was 0.84, which is close to that of the full model at 0.88. This indicates that the reduced model retained a significant portion of its explanatory power while using fewer variables. Moreover, the results of cross-validation using the ten-fold method supported this direction, as they showed that the reduced model achieved acceptable and comparable predictive performance to that of the full model, despite a slight increase in prediction error (1.37 vs. 1.21). This performance reflects the reduced model’s ability to generalize to new data, which adds practical value, especially in cases where reducing the number of variables and enhancing interpretability are among the main objectives of model construction.

**Fig 5:**
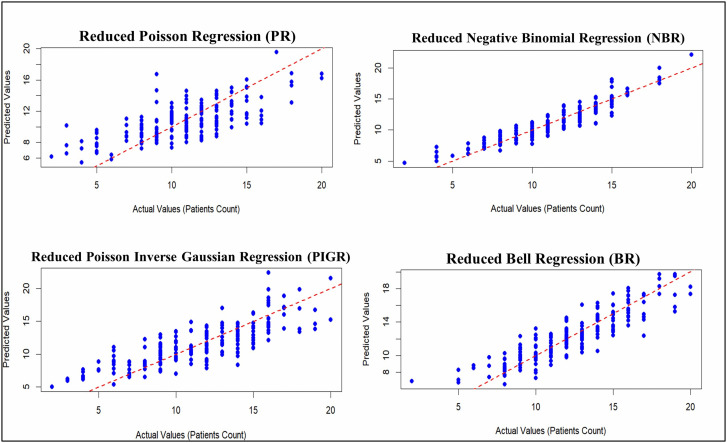
Plot of actual against predicted values for the reduced PR, NBR, PIGR, and BR models.

**Fig 6:**
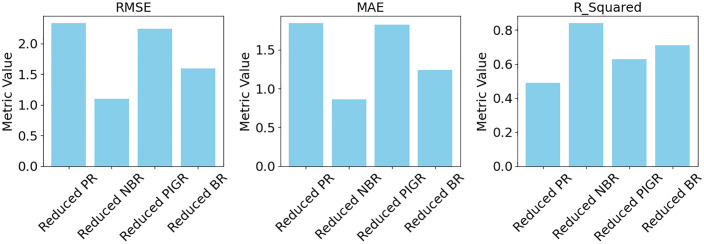
Model comparison across evaluation metrics.

**Fig 7:**
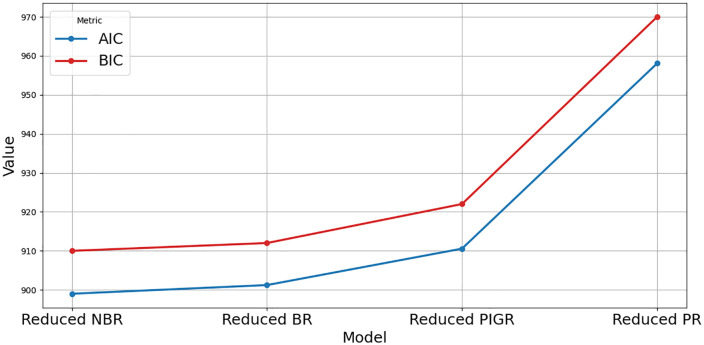
AIC and BIC comparison across reduced models.

Fig 5 presents the actual versus predicted values for the reduced models. The plots indicate that the reduced NBR models provide better predictive accuracy, with predicted values closely aligned with actual values. Figs 6 and 7 show a comparison of the evaluation metrics.

## 10 Likelihood Ratio Test to compare the performance of the reduced and full NBR model

To assess whether the fitness of the reduced NBR model is significantly different from the full NBR model, a Likelihood Ratio Test (LRT) was performed. The hypotheses tested were:

$H_{\text{0}}$= The reduced model fits the data as well as the full model.

$H_{\text{1}}$= The full model fits the data significantly better than the reduced model.

In this study, the log-likelihood values of the full and reduced NBR models were $log{\text{𝓁}}_{full}=\;-\text{418},\;log{\text{𝓁}}_{reduce}=-\text{423}$, leading to the LRT statistic:


LRT=−2[−423−[−418]]=10

The LRT statistic follows a chi-square distribution with degrees of freedom equal to the difference in the number of independent variables between the two models. In this case, the degrees of freedom were: df=18−5=13 and the p-value was 0.694.

Since the p-value is much greater than 0.05, we fail to reject the null hypothesis $H_{\text{0}}$. This indicates that there is no significant difference between the full and reduced models. Therefore, the reduced model, which includes only the five significant predictors (marital status, BMI, age, HDL, and TC), is capable of predicting the number of new cases with almost the same accuracy as the full model with 18 predictors. Consequently, the reduced NBR model is preferred due to its simplicity, parsimony, and nearly identical predictive power.

## 11 Discussion

This work presented in this paper is one of the most comprehensive studies carried out to predict the monthly number of new T2D cases in Saudi Arabia, utilizing local data and recommended key performance indicators (KPIs). De-identified data for 1,000 T2D patients between 2018 and 2022 were obtained from King Abdulaziz University Hospital (KAUH) in Jeddah, Saudi Arabia. The performance of the full and reduced Poisson Regression (PR), Negative Binomial Regression (NBR), Poisson Inverse Gaussian Regression (PIGR), and Bell Regression (BR) in predicting the monthly number of new T2D cases was compared based on their corresponding R², RMSE, MAE, CV-10, AIC, and BIC. The full models were developed using all the recommended KPIs, while the reduced models were built using only the most significant KPIs identified by all full models. The modeling approach was designed to reflect population-level trends, with all independent variables being monthly aggregated before building the model. We combined the data into monthly averages and proportions, which allowed us to capture overall patterns and understand how collective patient characteristics are associated with predicting the number of new T2D cases per month.

The results of the present study indicate a significant increase in new cases of T2D in Saudi Arabia between 2018 and 2022, with 2022 having the highest rate (see Table 1). The observations align with previously published work in Saudi Arabia, also indicated an increasing trend in the prevalence of T2D in the population, suggesting potential reasons for this increase, including urbanization, sedentary lifestyle, and high-energy diets [3,5,6]. In addition, similar increases in T2D incidence rates have been reported in other parts of the world, including China [18], the United States [19], and Bangladesh [21]. It has been demonstrated in prior works that PR, as well as NBR, models can be utilized for forecasting incidence rates [20,21].

The results of our analysis showed that the full NBR model outperformed the PR, PIGR, and BR models in predicting the monthly number of new T2D cases with R² = 0.88, RMSE = 0.93, MAE = 0.69, CV-10 = 1.21, AIC = 873.23, and BIC = 880. The most significant KPIs identified by all full models were marital status, age, total cholesterol (TC), body mass index (BMI), and high-density lipoprotein (HDL). Reduced models were developed using only these five KPIs. The reduced NBR model outperformed other reduced models with R² = 0.84, RMSE = 1.10, MAE = 0.86, CV-10 = 1.37, AIC = 899, and BIC = 910. Despite the higher AIC value of the reduced model compared with the full model (899 vs. 873.23), its performance remained highly competitive. The reduced model preserved most of the explanatory power of the full model while substantially improving model parsimony and interpretability.

Furthermore, the results of CV-10 confirmed that the predictive performance of the reduced model was acceptable and comparable to that of the full model, with only a marginal increase in prediction error (1.37 vs. 1.21). These findings justify the selection of the reduced NBR model as the most practical and efficient approach for predicting monthly T2D incidence, striking a balance between accuracy, simplicity, and generalizability. Moreover, the performance of full and reduced NRB was compared using the Likelihood Ratio Test (LRT). The results indicate no significant statistical difference between the full and reduced NBR. Therefore, the reduced NRB based on only five KPIs is recommended for predicting the number of new T2D cases each month.

Our analysis showed that BMI is one of the most significant KPIs associated with T2D, with individuals classified as overweight (BMI 25–29.9) or obese (BMI ≥ 30) showing a significantly higher incidence of T2D compared to those with normal weight, as shown in Table 1. It is notable that, although these findings conflict with some previous research [24,25], they are entirely consistent with other reports [18,19,21,23]. Additionally, the analysis revealed that marital status is a key variable associated with the prevalence of new cases of T2D. As shown in Table 1, 82% of T2D cases occurred in married individuals, which is a significantly higher percentage than that of unmarried individuals. This trend may be attributed to lifestyle changes after marriage, including increased caloric intake, reduced physical activity, and heightened psychosocial stress, all of which contribute to metabolic dysregulation. Furthermore, the patients showed abnormalities in the lipid profile. A high percentage (57.1%) had elevated levels of TC, highlighting the impact of dyslipidemia and noxious lipid accumulation on glucose metabolism. Additionally, 58.5% of patients exhibited decreased levels of HDL, a lipid fraction typically associated with metabolic protection. These findings emphasize the necessity of monitoring marital status, age, BMI, TC, and HDL levels when assessing the risk of T2D and developing targeted prevention strategies for the Saudi population.

This study has several strengths, the most notable of which is the use of a comprehensive set of recommended KPIs and multiple count regression models to predict the number of monthly new T2D cases in Saudi Arabia. We compared the performance of four full and reduced models (PR, NBR, PIGR, BR) using local data. The comparison between different count regression models presented in this paper provides valuable insights for future modeling of chronic disease surveillance, particularly in regions with a high prevalence of T2D. The steps outlined here can help researchers and public health authorities develop the most tailored model for their target population. The public health benefit of the study lies not only in predicting the population-level risk patterns but also in offering a predictive framework for estimating future monthly caseloads. These predictions are crucial for health system planning, resource allocation, and the design of targeted diabetes prevention and intervention strategies.

However, the study faced some limitations, the most significant of which was the lack of data on important factors such as genetic and hereditary factors. Some Saudi studies [49,50] have shown that omitting hereditary factors from predictive models may reduce their accuracy, particularly in high-risk populations like Saudi Arabia. Furthermore, studies in the Gulf region [51,52] confirm that family history significantly increases the risk of diabetes. Therefore, we recommend that future research integrate both genetic and clinical data to enhance understanding of the disease and improve the accuracy of the models. We also recommend the development of a nationwide electronic database system linking all hospitals in Saudi Arabia to improve nationwide data collection, sample size, and diversity.

## 12 Conclusion

This study represents one of the first comprehensive investigations to model the number of monthly new cases of Type 2 Diabetes (T2D) in Saudi Arabia using recommended Key Performance Indicators (KPIs) and multiple count regression models. Despite Saudi Arabia ranking among the countries with the highest prevalence of T2D globally, prior research in predicting the number of new cases of T2D has been limited. De-identified local data from 1,000 T2D patients has been utilized using four count regression models: Poisson Regression (PR), Negative Binomial Regression (NBR), Poisson Inverse Gaussian Regression (PIGR), and Bell Regression (BR) to predict the monthly number of new cases and identify the KPIs primarily associated with it.

The data collected showed a significant upward trend in the number of new T2D cases from 2018 to 2022, with the highest incidence observed in 2022. The comparison between the full models, which utilize all recommended KPIs, revealed that the full NBR model outperforms other models, achieving an R² = 0.88, RMSE = 0.93, MAE = 0.69, CV-10 = 1.21, AIC = 873.23, and BIC = 880. Marital status, age, total cholesterol (TC), body mass index (BMI), and high-density lipoprotein (HDL) were identified as significant KPIs by all models. The reduced NBR model based on only the five most significant variables achieved a predictive accuracy R² = 0.84. The results of the Likelihood Ratio Test (LRT) confirmed that there was no significant difference between the full and reduced models in predicting the number of monthly new cases of T2D. This performance reflects the reduced model’s ability to generalize to new data, which adds practical value, especially in cases where reducing the number of variables and enhancing interpretability are among the main objectives of model construction.

The findings highlight the significant role of specific demographic and biochemical factors in the development of T2D. These results align with global evidence linking obesity, dyslipidemia, and metabolic disturbances to the development of T2D.

The research outcomes can be used to develop targeted public health interventions and resource allocation strategies to reduce the disease burden and the monthly number of new T2D cases in the country by monitoring a much smaller number of recommended KPIs. The study also emphasizes the importance of establishing a unified and comprehensive health database to enhance data quality and model accuracy, ultimately supporting evidence-based healthcare strategies and improving population health outcomes. Furthermore, the findings of this study address a critical gap in T2D research in non-European countries and provide a foundation for future studies to explore additional risk factors and further enhance predictive models.
